# Methylation of the *SLC6a2* Gene Promoter in Major Depression and Panic Disorder

**DOI:** 10.1371/journal.pone.0083223

**Published:** 2013-12-02

**Authors:** Richard Bayles, Emma K. Baker, Jeremy B. M. Jowett, David Barton, Murray Esler, Assam El-Osta, Gavin Lambert

**Affiliations:** 1 Baker IDI Heart & Diabetes Institute, Melbourne, Australia; 2 St Vincent’s Institute, Melbourne, Australia,; CEA - Institut de Genomique, France

## Abstract

Reduced function of the noradrenaline transporter (NET) has been demonstrated in patients with major depressive disorder (MDD) and panic disorder. Attempts to explain NET dysfunction in MDD and panic disorder by genetic variation in the NET gene *SLC6a2* have been inconclusive. Transcriptional silencing of the *SLC6a2* gene may be an alternative mechanism which can lead to NET dysfunction independent of DNA sequence. The objective of this study was to characterise the DNA methylation state of the *SLC6a2* gene promoter in patients with MDD and panic disorder. *SLC6a2* promoter methylation was also analysed before and after antidepressant treatment. This study was performed with DNA from blood, using bisulphite sequencing and EpiTYPER methylation analyses. Patients with MDD or panic disorder were not found to differ significantly from healthy controls in the pattern of methylation of the SLC6a2 gene promotor. While significant correlations between methylation levels at some CpG sites and physiological measures were identified, overall the variation in DNA methylation between patients was small, and the significance of this variation remains equivocal. No significant changes in *SLC6a2* promoter methylation were observed in response to antidepressant treatment. Further in-depth analysis of alternative mechanisms of transcriptional regulation of the *SLC6a2* gene in human health and disease would be of value.

## Introduction

Reduced NET function has been demonstrated in patients with major depressive disorder (MDD), and panic disorder [1,2]. Extensive attempts to explain NET dysfunction in MDD and panic disorder by association with DNA sequence variation in the NET gene *SLC6a2* have been inconclusive, yielding weak association results and rare functional sequence variation [3]. The cause of NET dysfunction in these patient groups remains to be elucidated, however transcriptional silencing of the *SLC6a2* gene has been hypothesised. Promoter methylation has been demonstrated to correlate with many disease states, leading to reduced gene expression [4,5]. A previous report, based on pilot studies in leukocytes using methylation specific PCR (MSP), suggested that epigenetic silencing of the *SLC6a2* gene by methylation of the promoter CpG island may underly NET dysfunction in patients with panic disorder [6].. Based on these preliminary findings, a hypothesis was formed that with the development of panic disorder, the *SLC6a2* promoter is hypermethylated, leading to transcriptional repression of the *SLC6a2* gene. 

Importantly, MSP does not provide a quantitative measure of DNA methylation, provides severely limited coverage of CpG sites [7] and is not suitable for detecting subtle changes in DNA methylation which may be present in psychiatric diseases such as depression [7,8]. An unambiguous biological marker such as *SLC6a2* promoter methylation in a readily accessible tissue such as leukocytes would have high value in the diagnosis of complex diseases such as panic disorder and MDD. In light of these caveats a more in-depth investigation of *SLC6a2* promoter methylation in humans was required. From ongoing studies in our laboratory, genomic DNA was available for methylation analysis of human subjects for whom we had phenotypic evidence of NET dysfunction compared to healthy controls. 

A major advantage of peripheral tissues such as leukocytes is the ability to sample across the entire course of a disease. In addition to studying the epigenetic changes associated with the natural course of the disease, such as remission and relapse, samples can be analysed before, during and after treatment. In addition to untreated patients, DNA was extracted from some MDD and panic disorder patients before and after successful treatment with an SSRI for three months. The effect of SSRI treatment on DNA methylation in humans is unknown, however long-term changes in DNA methylation are hypothesised to be involved in the pathogenesis and remission of affective disorders [7,8]. 

The aim for this study was to investigate the methylation pattern of the *SLC6a2* promoter in detail, testing the hypothesis that an increase in *SLC6a2* promoter methylation in leukocytes may be a useful marker of NET dysfunction in human disease. Leukocytes from healthy subjects were compared with those from patients with MDD and panic disorder (pre- and post-treatment). Methylation analyses were performed using the gold standard bisulphite sequencing technique and EpiTYPER technology (Sequenom) [9]. 

## Methods

Human DNA samples were obtained from ongoing studies in MDD and panic disorder within the Human Neurotransmitters Laboratory at the Baker IDI Heart and Diabetes Institute as described previously [1,10]. All studies were approved by the Alfred Hospital Ethics Review Committee. All subjects gave written informed consent for participation including the use of their DNA in genetic studies. In the present study, DNA samples were obtained from blood from 70 healthy volunteers, 36 patients with MDD and 36 patients with panic disorder [10]. Catheter studies were not performed for the healthy patients. However, based on our previously published observations in healthy humans of cardiac extraction of tritiated noradrenaline being 80% ±1 % [1], both the MDD and panic disorder patients selected for this study had significantly reduced extraction of tritiated noradrenaline (56 % ±5 % and 72 % ±4 % respectively) across the heart, indicative of a defect in NET function [11,12]. DNA was also obtained from 5 MDD and 4 panic disorder patients mentioned above, both before and after treatment for three months with an SSRI (5 Citalopram, 2 Fluoxetine, 2 Sertraline). Patients and controls were excluded if they had coexisting heart disease, diabetes, medicated hypertension, alcohol or drug abuse or dependence, or infectious disease; had a comorbid psychotic disorder, eating disorder, mental retardation, personality disorder, or epilepsy; or had a current high suicide risk. Patients were either newly diagnosed or currently untreated after a relapse and had not been receiving antidepressants or benzodiazepines for at least 4 weeks prior to the study (5 weeks if they had been receiving fluoxetine hydrochloride). 

Differences between patient groups were determined by one-way ANOVA, with post-hoc multiple comparisons performed using the Holm-Sidak method. For data that was not normally distributed or with unequal variance, these data were ranked and Kruskal-Wallis one-way ANOVA performed with post-hoc multiple comparisons performed using Dunn’s method.

DNA was bisulphite converted, and promoter methylation was determined in 4 samples per group for untreated MDD, panic disorder and healthy controls by bisulphite sequencing covering 2 regions from −515 to −215 and −180 to +167 relative to the transcription start site (+1) using nested PCR as described previously [10]. These regions were selected based on our previous studies and unpublished data showing that these regions are transcriptionally important, contain multiple transcription factor binding sites, and beyond these regions outside the CpG islands of the promoter, DNA is typically methylated [13–15]. For one of the MDD patients in the bisulphite sequencing analysis, insufficient sample was available for analysis of both regions that were investigated, and this sample was replaced with a different MDD patient in the analysis of the second bisulphite sequencing region. The primers used for all PCRs are listed in Table 1, designed using the internet based program MethPrimer [16]. Differences in average methylation (%) for each region between subjects were determined by t-test. Some bisulphite sequencing data for 2 healthy controls as been published previously (Bayles et al, 2012).

**Table 1:** Bisulphite primer sequences for *SLC6a2*.

**Details**	**Sequence 5’-3’**
**Bisulphite sequencing**	
**Sense** Region 1, Round 1	ttattttgtttataaataatagag
**Anti-sense** Region 1, Round 1	cccaaacaaacctaaccctatccc
**Sense** Region 1, Round 2	gagttttttagatttttgggaatt
**Anti-sense** Region 1, Round 2	aaaaaatccctaataccttac
**Sense** Region 2, Round 1	gtaaggtattagggatttttt
**Anti-sense** Region 2, Round 1	atatattccaactcctatccca
**Sense** Region 2, Round 2	tggttttgggagttgtaagtag
**Anti-sense** Region 2, Round 2	aataaacctcccaaattcaaattcc
**EpiTYPER**	
**Sense** Region A	aggaagagagaggagtaagtgttgggttgtgattt
**Anti-sense** Region A	cagtaatacgactcactatagggagaaggctaattcccaaaaatctaaaaaactc
**Sense** Region B	aggaagagaggattttagttttggagagtttgttatt
**Anti-sense** Region B	cagtaatacgactcactatagggagaaggctcccaaacaaacctaaccctatcc

### EpiTYPER methylation analysis

Promoter methylation of the *SLC6a2* gene was also investigated using EpiTYPER methylation analysis on the MassARRAY® platform (Sequenom). Patient details are provided in Table 2. In preparation for EpiTYPER methylation analysis, DNA was bisulphite converted and primers were designed for multiple regions using reference sequences of the human *SLC6a2* gene promoter (NCBI sequence accession numbers AF061198 and NC_000016). Primers for EpiTYPER analyses were provided by Sequenom (San Diego, CA, USA). EpiTYPER methylation analysis was performed according to standard manufacturer instructions including both C and T cleavage reactions (User Guide Version 1.0). Data was analysed using EpiTYPER Version 1.2 software (Sequenom). 

**Table 2:** Patient data for methylation analysis.

**Pathology**	**n**	**Age**	**Sys BP**	**Dia BP**	**HR**	**BMI**	**Trait**	**State**	**[NA]**	**[DHPG]**	**NA:DHPG**
Control	70 (47M/23F)	39 ±2	129 ±2	73 ± 2	65 ± 1	25 ± 1	30 ± 2	32 ± 2	220 ± 18	1480 ± 158	7.4 ± 0.9
MDD	36 (18M/18F)	42 ±2	133 ±3	71 ± 2	66 ± 2	26 ± 1	*63 ± 2	*57 ± 4	297 ± 38	1285 ± 169	5.4 ± 0.7
PD	36 (18M/18F)	37 ±2	140 ±4	72 ± 2	*73 ± 2	26 ± 1	43 ± 5	*42 ± 4	272 ± 26	1169 ± 120	*4.8 ± 0.5

All data are from first visit (untreated)

^*^ p<0.05, significantly different to healthy control group. Data presented are the means ± SEM

Sys BP & Dia BP, systolic & diastolic blood pressure (mmHg); HR, heart rate; BMI, body mass index; Trait and State, Spielberger’s anxiety scores; [NA], arterial noradrenaline concentration (pg/mL); [DHPG], arterial DHPG concentration (pg/mL)

EpiTYPER methylation data were annotated as described in the publication by Coolen et al. (2007) to highlight which data are averages from multiple CpG sites. A period (.) between numbers denotes CpG sites within the one fragment [17]. As an example: CpG 19.20.21, illustrates that CpG sites 19, 20 and 21 are in the same fragment. Data are presented as mean methylation + SEM for each CpG site / fragment. The legend in each graph represents the subject group for which the average methylation was determined. 

Statistical analysis was performed using SigmaStat Version 3.5 (Systat Software, Point Richmond, CA). Analysis of the level of methylation at each CpG site across groups was performed using two-way ANOVA. Associations between the degree of methylation at the various sites and demographic and physiological variables were examined using forward and backward stepwise multiple regression analysis with cumulative R^2^ values shown. A value of P<0.05 was considered significant.

## Results

The *SLC6a2* promoter was analysed from −515 to +167 in two regions using bisulphite sequencing, and two EpiTYPER regions which corresponded approximately with the two bisulphite sequencing regions. Anxiety scores were higher in patients with MDD and panic disorder, and heart rate was higher in panic disorder patients (Table 2). The arterial DHPG:NA (dihydroxyphenolglycol : noradrenaline) plasma ratio was significantly lower in panic disorder, an additional indication of a defect in noradrenaline uptake.

### Bisulphite sequencing of the *SLC6a2* promoter in MDD and panic disorder

**Figure 1:**
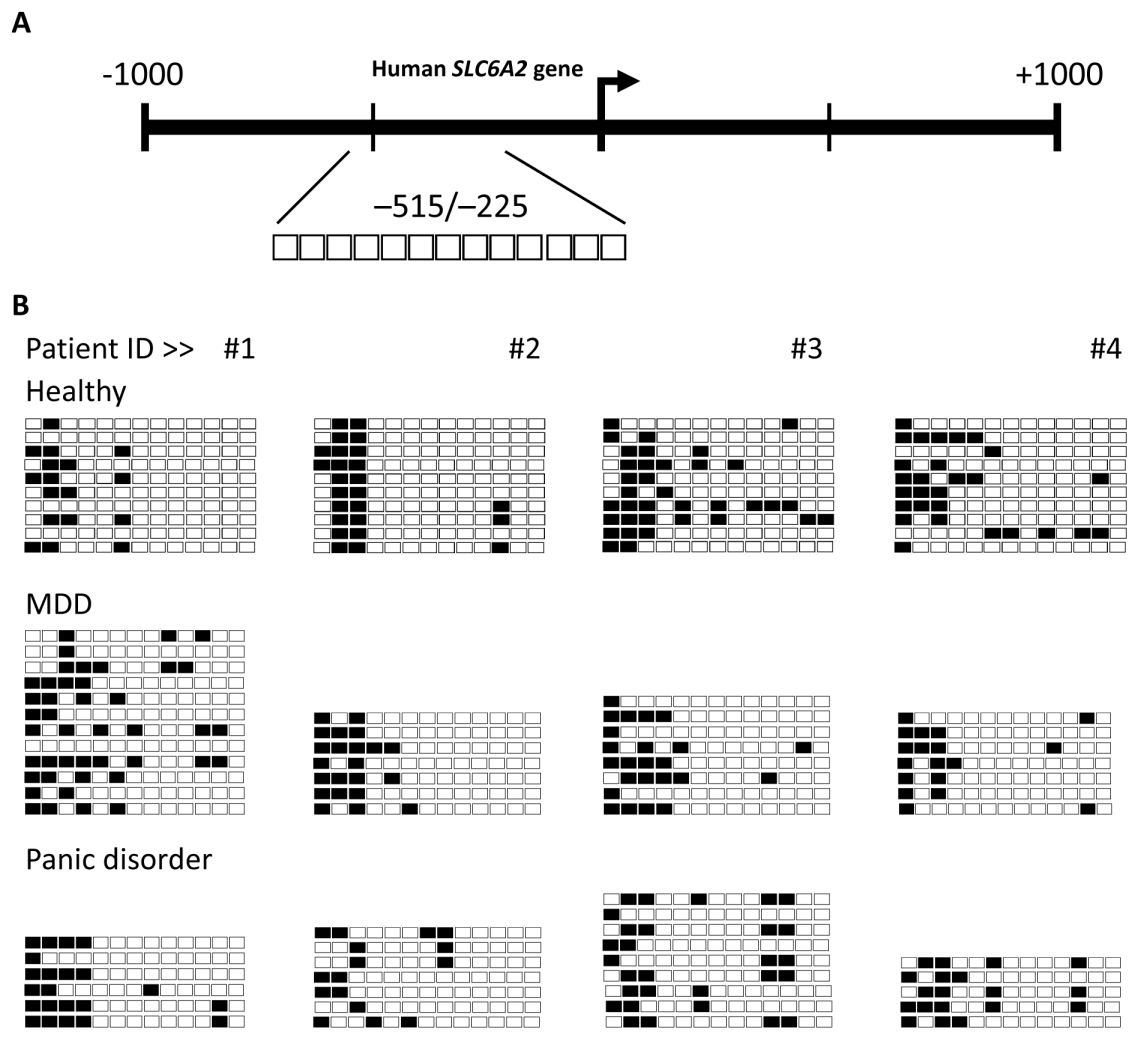
CpG methylation in Region 1 of the *SLC6a2* promoter in humans. The methylation status of *SLC6a2* promoter Region 1 (-515 bp to -225 bp) in leukocytes was analysed by bisulphite sequencing in 4 healthy subjects compared to 4 subjects with MDD or panic disorder (#1-4). **A**) A schematic representation of Region 1. The individual CpG sites analysed in Region 1 are indicated as enlarged boxes with their positioning relative to the transcription start site (+1) indicated with an arrow. **B**) Bisulphite sequencing data for MDD, panic disorder and healthy controls. Each row of boxes represents a single cloned allele, and each box represents a single CpG dinucleotide. Black boxes indicate methylated cytosine residues, white boxes indicate unmethylated cytosine residues. For healthy subjects #1-4, 10 individual clones were sequenced. For MDD subjects #1-4, between 7 and 12 individual clones were sequenced. For panic disorder subjects #1-4, between 5 and 9 individual clones were sequenced for each subject.

The *SLC6a2* promoter methylation within Region 1 was analysed in four untreated subjects per group. In all groups, the CpG dinucleotides located outside of the CpG island defined by Methprimer (in the 5’ end of Region 1) were hypermethylated. In contrast, CpG dinucleotides within the CpG island (towards the 3’ end of Region 1) were hypomethylated in all subjects (Figure 1). Although some individual CpG sites were identified to be methylated in this region, the methylation status was not consistent between subjects or multiple alleles in a single subject. No statistically significant pattern was identified in an individual, or in a particular subject group when examining the average percentage methylation of the region between groups.

**Figure 2:**
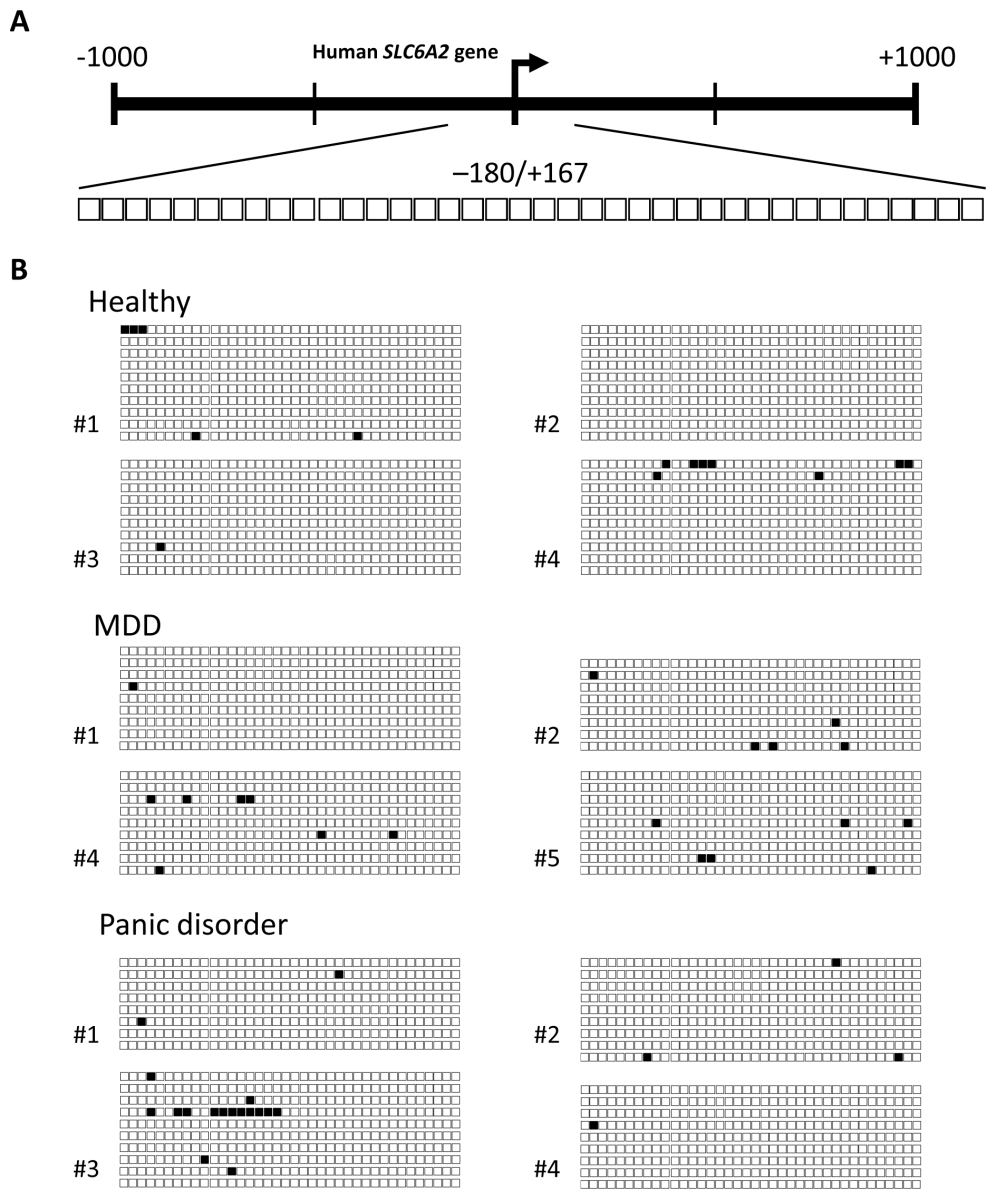
CpG methylation in Region 2 of the *SLC6a2* promoter in humans. The methylation status of the *SLC6a2* promoter Region 2 (-180 bp to +167 bp) was analysed by bisulphite sequencing in 4 healthy subjects compared to 4 subjects with MDD and panic disorder. **A**) A schematic representation of Region 2. The individual CpG sites analysed in Region 2 are indicated as enlarged boxes with their positioning relative to the transcription start site (+1) indicated with an arrow. **B**) Bisulphite sequencing data are presented as in Figure 1. For healthy subjects #1-4, 10 individual clones were sequenced. For MDD subjects #1-4, between 8 and 9 individual clones were sequenced. A different MDD patient (#5) replaced sample #3 due to insufficient sample. For panic disorder subjects #1-4, between 8 and 10 individual clones were sequenced.

*SLC6a2* promoter methylation within Region 2 was analysed in the same four subjects from each group except for MDD patient 5 which replaced #3 due to insufficient DNA for analysis. Region 2 encompasses a dense region of the first CpG island in the promoter (38 CpG sites), and includes 4 putative Sp1 transcription factor binding sites, of which 3 contain CpG dinucleotides within their consensus site. In contrast to Region 1 which showed a heterogeneous methylation pattern between all groups, Region 2 was almost completely unmethylated in all subjects examined (Figure 2).

The pattern of hypomethylation observed in Region 2 was quite homogenous between patients and healthy controls. Region 1 however, exhibited more heterogeneity between patients, making it difficult to detect differences between groups. With a higher throughput method such as EpiTYPER analysis, issues such as the need for higher numbers of clones to achieve more accurate methylation percentages, and more patient numbers to control for intra-group variation could be addressed.

### EpiTYPER methylation analysis of the *SLC6a2* promoter in MDD and panic disorder

EpiTYPER regions were designed and labelled Regions A and B so as to differentiate them from bisulphite sequencing Regions 1 and 2. EpiTYPER Region A corresponded to bisulphite sequencing Region 1, and Region B corresponded to bisulphite sequencing Region 2. All patient and healthy control samples analysed by bisulphite sequencing were included (except for MDD patient 3). 

**Figure 3:**
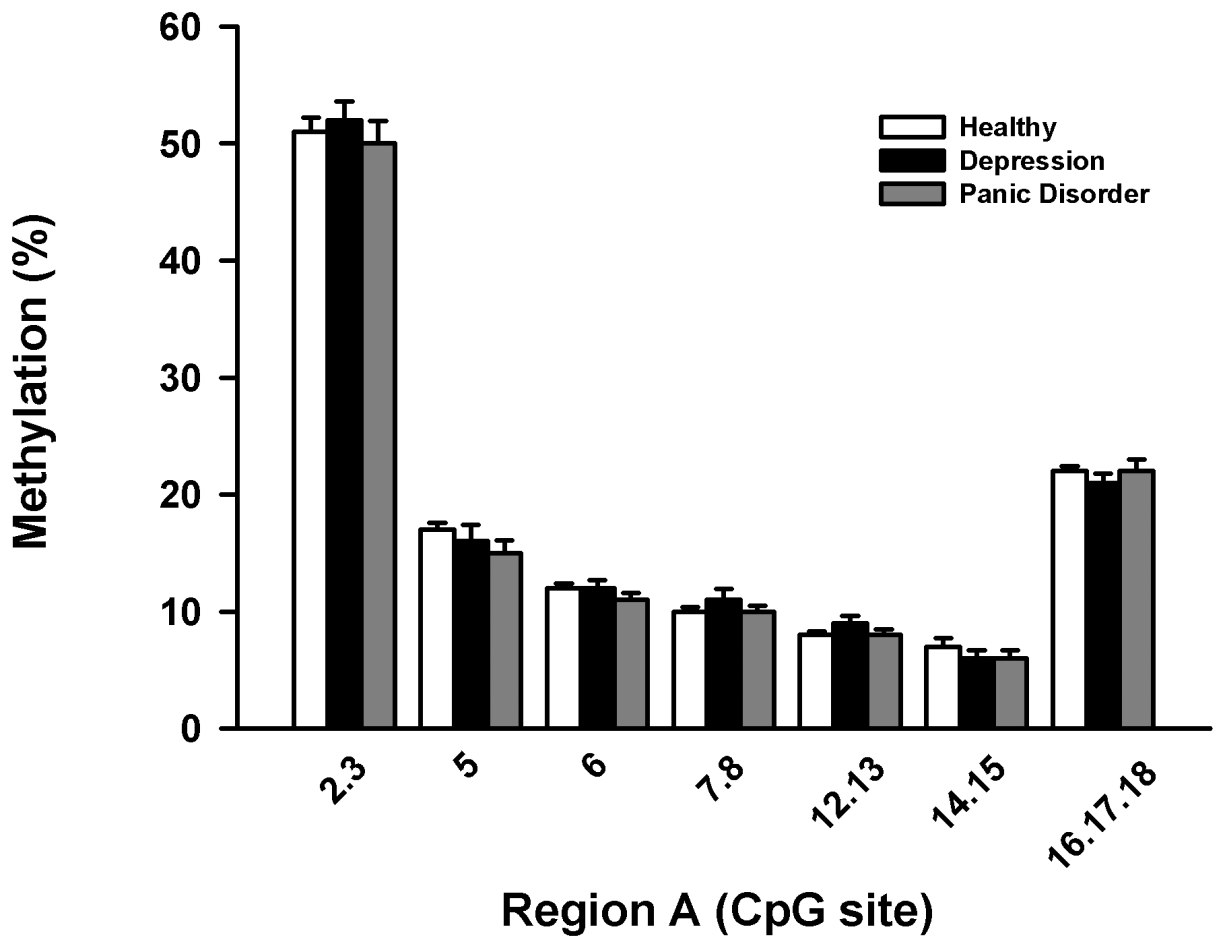
CpG methylation of Region A of the human SLC6a2 promoter. Representation of the methylation percentages for CpG sites in Region A of the *SLC6a2* gene determined by EpiTYPER analysis. CpG site. CpG sites are numbered 5’- 3’ along the X-axis, with percentage methylation on the Y-axis. Usable data could not be obtained for all CpG sites for all samples. All groups exhibited a similar methylation profile across the region, and no difference between groups for the region as a whole was determined to be significant by two-way ANOVA.

**Figure 4:**
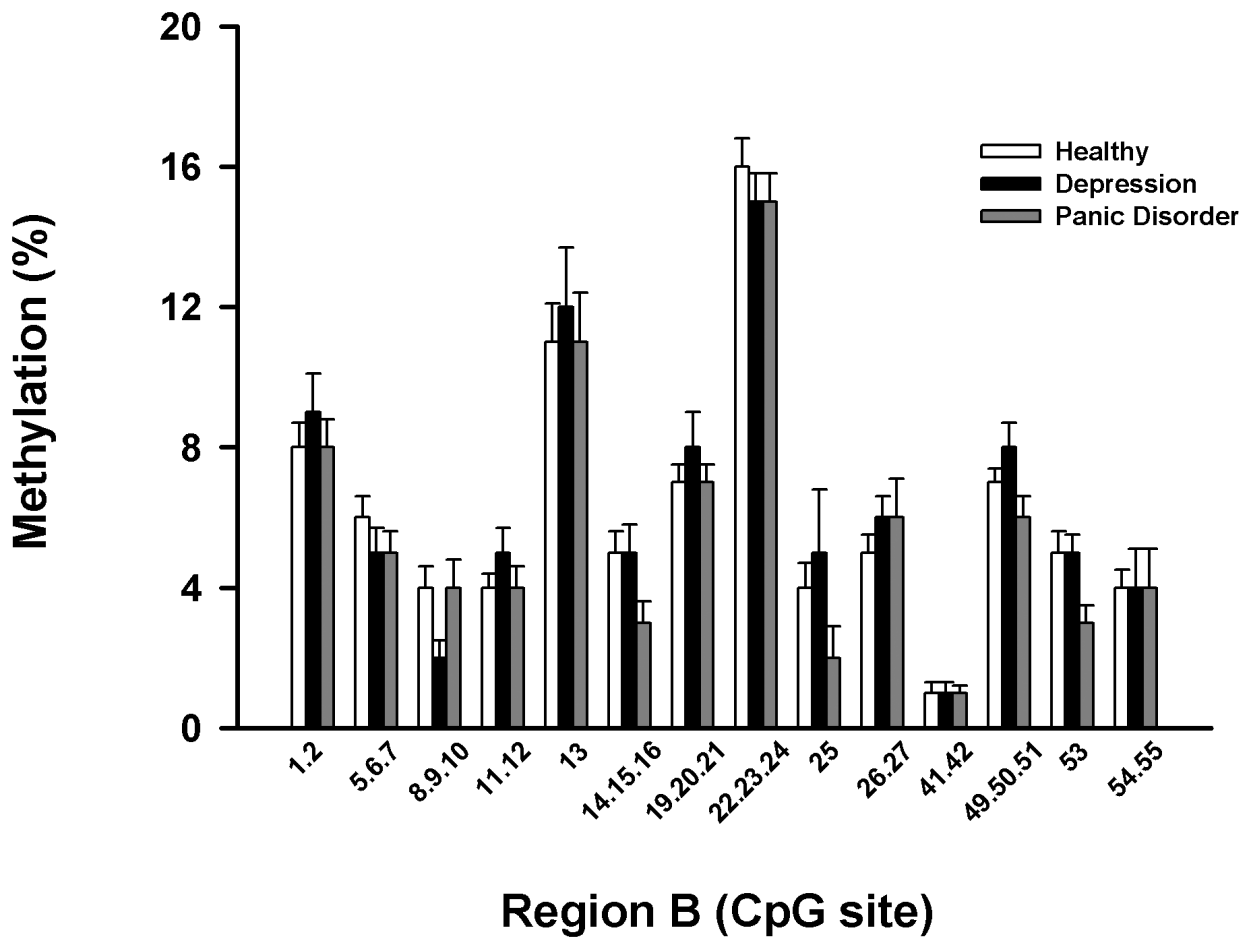
CpG methylation of Region B of the human SLC6a2 promoter. Representation of the methylation percentages for CpG sites in Region B of the *SLC6a2* gene determined by EpiTYPER analysis. CpG sites are numbered 5’- 3’ along the X-axis, with percentage methylation on the Y-axis. Usable data could not be obtained for all CpG sites for all samples. The overall methylation of Region B in subjects with MDD was determined to be significantly greater than all other groups (p<0.05).

EpiTYPER methylation data for all groups for Regions A and B are presented in Figure 3 & Figure 4. Statistical analyses of the pooled averages of sample groups was performed based on previous reports which have used EpiTYPER arrays to determine methylation differences between sample groups [18,19]. Over the two regions analysed, the average methylation of any given CpG site did not differ between patient groups and healthy controls by a magnitude greater than 5 %, the technical standard deviation of the EpiTYPER technique. The methylation levels of the *SLC6a2* promoter as a whole in Regions A and B analysed using the EpiTYPER method were not significantly different between subjects with MDD or panic disorder and healthy controls (Figures 3&4). The methylation across the regions analysed by EpiTYPER assays was generally consistent between all sample groups, and with low variation within groups. The overall pattern of hypomethylation across CpG dense regions of the promoter was consistent with the bisulphite sequencing results.

Despite the small differences in average methylation between groups, when all methylation data was combined and correlated with basic physiological measures associated with NET function, some significant associations were identified. For Region A, forward stepwise regression analysis revealed that diastolic blood pressure could be predicted by the level of methylation at sites 14 and 15 (P = 0.01) and 7 and 8 (P = 0.02), with the combination of these factors accounting for 14.5% of the variance in diastolic blood pressure (Table 3). Methylation at other sites examined did not significantly add to the ability to predict diastolic blood pressure. There was no association between the degree of methylation at other sites on Region A and any other physiological variable including age, BMI and gender.

**Table 3:** Association between Region A methylation and diastolic blood pressure.

**Step**	**Methylation site**	**R**	**Cumulative R^2^**	**Delta R^2^**
1	14.15	0.29	0.087	0.087
2	7.8	0.38	0.145	0.058

Significant correlations between DNA methylation in Region A and physiological measures detailed in Table 2 in all participants combined

For Region B there occurred an association between age and degree of methylation at sites 22, 23, 24, 14, 15, 16, 8, 9 and 10 (P = 0.007 for the model). After controlling for age and gender, BMI was related to the degree of methylation at sites 8, 9 and 10 (R = 0.22; P = 0.03), and systolic blood pressure could be predicted by the level of methylation at sites 53 and 13 (P=0.006 for model), with the combination accounting for 13.3% of the variability in systolic blood pressure (Table 4). Diastolic blood pressure was related to methylation at sites 54 and 55 (P =0.02) whilst heart rate could be predicted by the degree of methylation at sites 1, 2, 13, 26 and 27 (P=0.005 for the model). Arterial noradrenaline levels were related to methylation at sites 26 and 27 (R = 0.37, P = 0.004) whilst the arterial DHPG concentration could be predicted from a linear combination of methylation at sites 13, 25, 26 and 27.

**Table 4:** Association between Region B methylation and physiological variables.

**Variable**	**Step**	**Methylation site**	**R**	**Cumulative R^2^**	**Delta R^2^**
Age	1	22.23.24	0.22	0.050	0.050
	2	14.15.16	0.31	0.098	0.048
	3	8.9.10	0.36	0.132	0.034
BMI	1	8.9.10	0.22	0.05	0.05
Systolic BP	1	53	0.25	0.060	0.060
	2	13	0.37	0.133	0.073
Diastolic BP	1	54.55	0.26	0.065	0.065
Heart Rate	1	1.2	0.29	0.082	0.082
	2	26.27	0.37	0.134	0.051
	3	13	0.43	0.187	0.054
Arterial NA	1	26.27	0.37	0.134	0.134
Arterial DHPG	1	13	0.46	0.208	0.208
	2	25	0.55	0.302	0.094
	3	26.27	0.62	0.381	0.079

Significant correlations between DNA methylation in Region B and physiological measures detailed in Table 2 in all participants combined. BMI, body mass index; BP, blood pressure; NA, noradrenaline; DHPG, dihydroxyphenolglycol

### *SLC6a2* promoter methylation and selective serotonin reuptake inhibition

**Figure 5:**
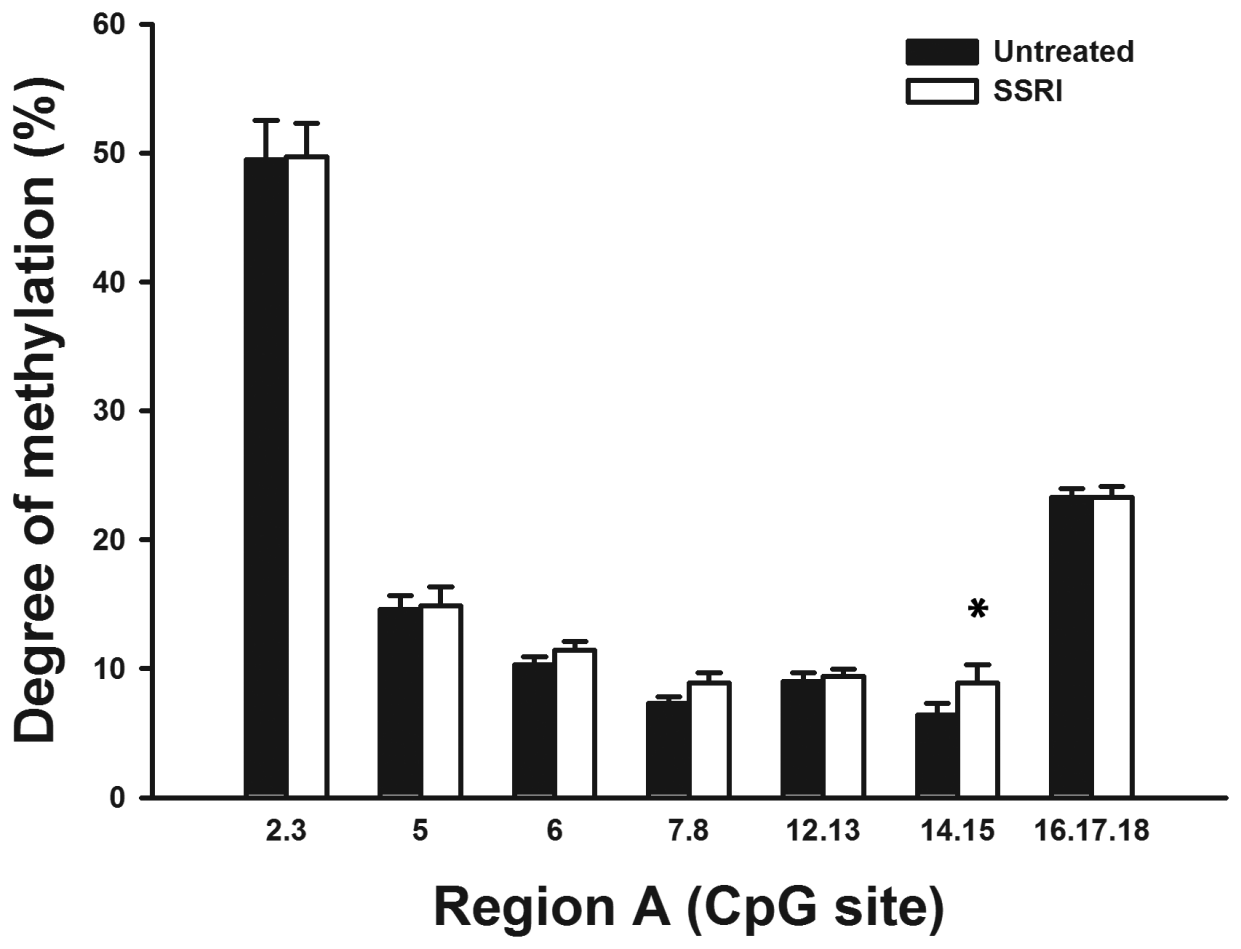
Analysis of methylation in Region A pre- and post-SSRI treatment. Representation of the methylation percentages for CpG sites in Region A of the NET gene determined by EpiTYPER analysis in human leukocytes pre- and post-SSRI treatment. CpG sites are numbered 5’- 3’ along the X-axis, with percentage methylation on the Y-axis. Usable data could not be obtained for all CpG sites for all samples. Samples pre- and post-SSRI treatment exhibited a similar methylation profile across the region, and no differences in average methylation of CpG sites in response to SSRI treatment were determined to be significant by paired t-test. *Statistically significant site-specific difference in methylation with SSRI treatment.

**Figure 6:**
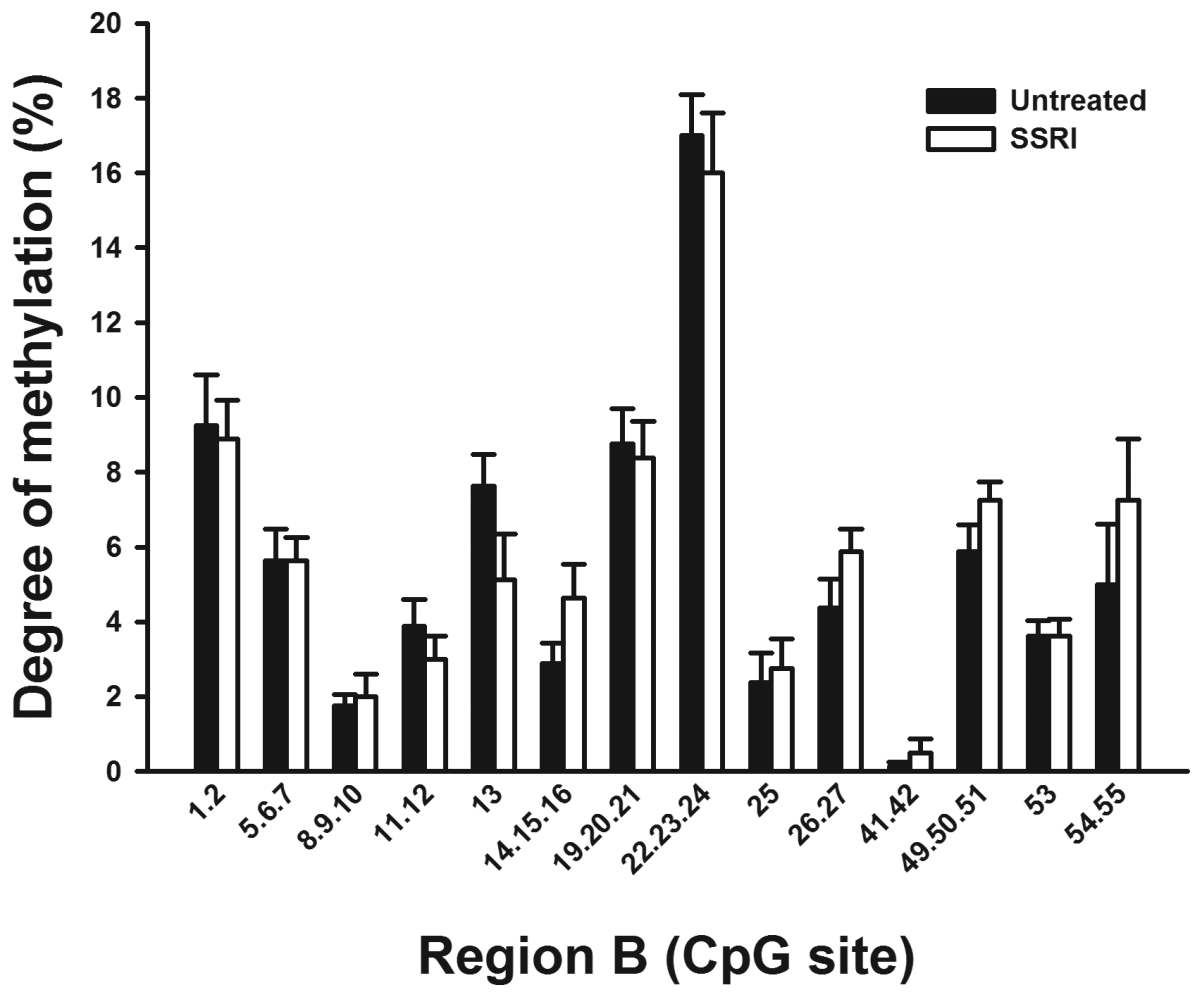
Analysis of methylation in Region B pre- and post-SSRI treatment. Representation of the methylation percentages for CpG sites in Region B of the NET gene determined by EpiTYPER analysis in human leukocytes pre- and post-SSRI treatment. CpG sites are numbered 5’- 3’ along the X-axis, with percentage methylation on the Y-axis. Usable data could not be obtained for all CpG sites for all samples. Samples pre- and post-SSRI treatment exhibited no significant difference in methylation profile across the region.

For 5 subjects with MDD and 4 with panic disorder, DNA samples were available before and after treatment with an SSRI for three months. Samples pre- and post-treatment were analysed to determine if methylation of the *SLC6a2* promoter in leukocytes changed in response to SSRI treatment (Figures 5 & Figure 6). MDD and panic disorder subjects were grouped for this analysis. A statistical difference in the methylation of specific CpG sites in Region A was determined, (there were no additional differences when analysing individual patient groups separately). Sites 14 and 15 were found to have significantly higher methylation after SSRI treatment.

## Discussion

### Methylation of the *SLC6a2* gene promoter

The *SLC6a2* gene has been hypothesised to be repressed in human diseases where evidence of NET dysfunction exists due to an increase in DNA methylation of the *SLC6a2* gene promoter [6,21]. The regulatory importance of methylation of CpG islands in the repression of gene expression has long been established [22], and repeatedly demonstrated in cancer [23]. The original studies which reported increased *SLC6a2* promoter methylation in hypertension and panic disorder were performed in leukocytes [6,21]. Using the same tissue source and bisulphite sequencing and Sequenom’s EpiTYPER technology however, the CpG island that encompasses the *SLC6a2* transcription start site was demonstrated to be generally hypomethylated in leukocytes of healthy controls and patient groups, with or without SSRI treatment in this study. This result is consistent with what we previously observed in patients with the postural tachycardia syndrome in whom there existed clinical and functional evidence of a defect in NET function [10]. Methylation patterns in the two regions covered by both bisulphite sequencing and EpiTYPER, were consistent between methodologies. 

The human *SLC6a2* gene has a CpG island within the proximal promoter [10]. The region around the transcription start site is particularly dense, and contains multiple putative transcription factor binding sites. The relevance of this is highlighted by the methylation state of Sp1 transcription factor binding sites, for example, being shown to alter the binding of Sp1, affecting gene expression [24,25]. The *SLC6a2* gene has multiple putative Sp1 binding sites in its promoter and the methylation status of four of these sites were not different between groups. The methylation levels of several CpG sites were found to positively correlate significantly with blood pressure, heart rate, age, BMI and arterial concentrations of noradrenaline and its metabolite DHPG. In Region B, CpG 13 which correlated with blood pressure, heart rate and DHPG, and CpG 25 which correlated with DHPG levels, correspond with the binding sites for the transcription factors Sp1 and serum response factor (SRF) respectively [20]. The methylation status of these CpG sites could potentially affect the binding of transcription factor complexes that associate with the SRE, consequently reducing NET expression. This hypothesis is analogous to the potential for SNPs within SREs to affect *SLC6a2* gene transcriptional efficiency as has previously been reported [26]. This potential mechanism requires further study. DNA methylation variance was low in this study, and must be considered in context of the precision of the EpiTYPER technology, which is a reported sensitivity of an ability to detect methylation differences of 5 % or greater (17). However, while the magnitude of differences in methylation in this study may be considered small, methylation changes of a similar scale in the hypomethylated promoters of three stress-related genes in mice have been associated with significant changes in gene expression [27]. 

Why the methylation of the *SLC6a2* promoter may correlate with age, and physiological markers including blood pressure and heart rate for example, may be explained to some extent by recent studies focussed on the effects of stress and aging on the DNA methylome. A number of studies have recently demonstrated significant divergence of methylation patterns with aging, and in conjunction with stress exposure [28–30]. We could not differentiate between healthy controls and patient groups with stress related disorders and significantly higher levels of anxiety however, which suggests that age may actually be the more important factor determining the methylation status of the *SLC6a2* promoter. It may be interesting to explore these correlations further in a healthy aging cohort, and also in hypertension to explore the blood pressure effect. Healthy aging and hypertension are both associated with a decrease in NET function [12,31,32].

An altered pattern of methylation in patients could ultimately affect the chromatin structure and the binding profile of transcriptional machinery that interacts with the *SLC6a2* gene promoter, consequently altering expression of the *SLC6a2* gene. The hypothesis that the differences in DNA methylation may affect *SLC6a2* gene expression remains to be tested however. This may be achieved in future using a cell-based model in which *SLC6a2* gene transcription can be measured, and the methylation of the *SLC6a2* promoter can be manipulated, and proteins binding to the *SLC6a2* gene can be analysed by chromatin immunoprecipitation (ChIP) assays. 

Increased noradrenaline spillover has been described in some subjects with MDD, and upon successful treatment with an antidepressant (selective serotonin reuptake inhibitor; SSRI) for three months, noradrenaline spillover levels were reduced in these patients [1]. While long term gene expression changes in response to antidepressant treatment have been hypothesised [33], studies on the effects of antidepressants on DNA methylation are limited. However, a role for histone modifications in the action of some antidepressant treatments and electroconvulsive seizures (a form of treatment for MDD) has previously been demonstrated [34,35]. Experiments in mice have also demonstrated that SSRI treatment causes the induction of methyl-DNA binding proteins [36], however the potential affect of SSRI treatment on DNA methylation in humans remains unknown. The ability of the SSRI fluoxetine to alter the expression of proteins with methyl-CpG binding domains (MBDs) has been reported [36]. The average methylation of the *SLC6a2* promoter in this study between subjects with MDD and healthy controls were not changed by SSRI treatment however. While a statistically significant increase in DNA methylation was determined for CpG sites 14 and 15 in Region A, the levels of methylation were already too low to determine accurately by the EpiTYPER methodology, and the magnitude of change also too small. In this context we can not conclude that we have identified a change in DNA methylation of the *SLC6a2* promoter in response to SSRI treatment in our study. Treatment of MDD with an SSRI was shown previously not to affect tritiated noradrenaline uptake across the heart [1]. The effects of antidepressants on methylation of the *SLC6a2* gene could be tested more thoroughly using cell-based systems, animal models or post-mortem human tissues, comparing tissues from subjects with and without MDD, and with or without antidepressant treatment. 

It was hypothesised that genomic DNA from human leukocytes may be useful in a search for peripheral changes in DNA methylation, reflecting epigenetic differences on the *SLC6a2* promoter in other tissues in various disease states. Expression of NET mRNA could not be detected in human leukocytes however to allow a correlation to be made with methylation profiles, and while sampling of sympathetically innervated hand and forearm veins is possible, these sites are far removed from the sympathetic nerve cell nuclei and are unsuitable for measurement of NET mRNA [6]. Although NET mRNA could not be detected in leukocytes, decreased expression of NET protein has been reported in peripheral tissues. In leukocytes in patients with depression, reduced binding of nisoxetine was detected compared to healthy controls [37]. As nisoxetine selectively bind to NET, this data was proposed to represent a reduction in NET expression in these cells, however measurement of nisoxetine binding may only reflect the NET protein presented at the cell surface, not the total expression level. 

The small differences in methylation detected in leukocytes in this study should be treated with some caution, not only in terms of the potential biological relevance of such small changes. Leukocytes are a heterogeneous cell population, and methylation differences detected in these samples may reflect changes in a specific subset of cells. It may be possible to characterise cell-type specific methylation patterns in leukocytes using cell sorting techniques, however this was not performed in this study. MDD for example, has been associated with systemic inflammation and SSRI therapy linked with increased levels of hs-CRP [38–40]. Such scenarios may alter the composition of cell types in the leukocyte extracts from these patients potentially skewing the average DNA methylation profile in unsorted leukocytes. In a study by Byun et al. (2009), DNA methylation patterns between different people, and between different tissues within an individual were found to be highly conserved [41]. Despite *SLC6a2* gene methylation patterns in leukocytes potentially being consistent with those observed in other tissues, it can not be ruled out that DNA methylation may differ specifically in diseased sympathetic nerve nuclei. 

This study analysed leukocyte samples from human subjects and found the *SLC6a2* promoter to be hypomethylated. MeCP2 binding was originally believed to be dependent on DNA methylation, although recent studies including our own, have established that this is not always the case [10,15]. We have previously demonstrated that NET expression can be regulated independently of promoter DNA methylation, by mechanisms involving the binding of MeCP2 and modification of histone tails [13,15]. With the recent discovery of 5-hydroxymethylation (5hmC) being particularly abundant in the nervous system, and associated with the euchromatin of active genes, the possibility also exists now for characterisation of the potential role of 5hmC in the regulation of the *SLC6a2* gene [42]. In a background of limited success of genetic association studies, it is possible that some single nucleotide polymorphisms (SNPs) in the *SLC6a2* gene locus that are functionally relevant may have been overlooked. That is to say that SNPs altering CpG sites may now need to be considered not only in the context of DNA methylation but also hydroxymethylation. Future in-depth investigation of such factors, in parallel with MeCP2 binding and histone modifications [13,15] as an alternative mechanism of *SLC6a2* gene regulation in MDD and panic disorder is warranted.
